# Realization of high-luminous-efficiency InGaN light-emitting diodes in the “green gap” range

**DOI:** 10.1038/srep10883

**Published:** 2015-06-03

**Authors:** Yang Jiang, Yangfeng Li, Yueqiao Li, Zhen Deng, Taiping Lu, Ziguang Ma, Peng Zuo, Longgui Dai, Lu Wang, Haiqiang Jia, Wenxin Wang, Junming Zhou, Wuming Liu, Hong Chen

**Affiliations:** 1 Key Laboratory for Renewable Energy, Beijing Key Laboratory for New Energy Materials and Devices, Beijing National Laboratory for Condensed Matter Physics, Institute of Physics, Chinese Academy of Sciences, Beijing 100190, China

## Abstract

Light-emitting diodes (LEDs) in the wavelength region of 535–570 nm are still inefficient, which is known as the “green gap” problem. Light in this range causes maximum luminous sensation in the human eye and is therefore advantageous for many potential uses. Here, we demonstrate a high-brightness InGaN LED with a normal voltage in the “green gap” range based on hybrid multi-quantum wells (MQWs). A yellow-green LED device is successfully fabricated and has a dominant wavelength, light output power, luminous efficiency and forward voltage of 560 nm, 2.14 mW, 19.58 lm/W and 3.39 V, respectively. To investigate the light emitting mechanism, a comparative analysis of the hybrid MQW LED and a conventional LED is conducted. The results show a 2.4-fold enhancement of the 540-nm light output power at a 20-mA injection current by the new structure due to the stronger localization effect, and such enhancement becomes larger at longer wavelengths. Our experimental data suggest that the hybrid MQW structure can effectively push the efficient InGaN LED emission toward longer wavelengths, connecting to the lower limit of the AlGaInP LEDs’ spectral range, thus enabling completion of the LED product line covering the entire visible spectrum with sufficient luminous efficacy.

Light-emitting diode (LED), one of the most important optoelectronic devices, has been used in a wide range of applications^1^,^2^,^3^, such as display backlights and general illumination. Great progress^4^,^5^,^6^,^7^ has been made since the first candela-class high-brightness InGaN-based LED was realized in 1994 by Shuji Nakamura^8^. A high external quantum efficiency (EQE) of 80% is achieved^9^ for InGaN blue LEDs; however, for emissions with wavelengths beyond 535 nm, the EQE of InGaN-based LEDs decreases dramatically as the wavelength increases^10^. AlGaInP LEDs, which have a high efficiency when emitting red and yellow light, are not suitable for wavelengths shorter than 570 nm because of the direct-indirect bandgap transition when the aluminum molar concentration reaches approximately 53%^11^.Commercialized LEDs with emission wavelengths between 535 and 570 nm to match the highest spectral sensitivity of the human visual perception of brightness are still absent, which is known as the “green gap” problem^12^.

Because the bandgap of InGaN covers the wide spectral range from near ultraviolet to near infrared, it is possible to realize high-brightness light emission for wavelengths greater than 535 nm. The problem is that the efficiency of InGaN LEDs typically decreases rapidly with the increasing indium content required to achieve longer-wavelength emissions. Generally, two main factors are responsible for the efficiency decrease: (1) the crystal quality deterioration due to the lattice mismatch between InGaN and GaN and indium phase separation^13^ and (2) the reduction of the electron-hole wavefunction overlap due to the strong polarization field in InGaN/GaN MQWs, which is also known as the quantum-confined Stark effect^14^ (QCSE). Various approaches^15^,^16^,^17^,^18^,^19^,^20^,^21^ have been proposed to overcome these problems. The growth conditions of InGaN have been optimized to reduce defects in the InGaN active region. Additional processes, including indium pre-deposition before InGaN growth^15^, as well as multiple growth interruptions during the quantum well (QW) growth^16^, are reported to enhance InGaN quality. To control the QCSE, InGaN/GaN MQW structures have been grown on nonpolar and semipolar GaN substrates, and a bright green light with an output power of 9.9 mW was obtained at 20 mA on semipolar 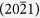 bulk GaN substrates^17^. Because only c-plane GaN is used in the industrial manufacture of LEDs, control of the QCSE of LEDs on c-plane sapphire substrates is significant, and this control has been explored by QW band engineering approaches, including the staggered QW structure^18^, triangular-shaped QW^19^, type-II InGaN QW^20^, an embedded AlGaN layer^21^, and so on. All of these structures confine the carriers to enlarge the overlap of the electron-hole wavefunctions. A high output power of 11.0 mW for a 559-nm LED at a 20-mA injection current and 5.71-V forward voltage (LED products require lower than 3.6 V) were achieved by inserting an AlGaN layer between the QW layer and upper barrier layer^21^. Here, we report high-output-power “green gap” InGaN LEDs operating with a normal voltage on c-plane sapphire substrates. The InGaN/GaN MQWs in the active region have a hybrid design; specifically, the active region is composed of one high-indium QW in the “green gap” range near the p-type GaN side on top of four low-indium QWs in the blue wavelength range near the n-type GaN side. We prove that the harmful effects of the crystal quality deterioration and the QCSE are reduced by the stronger localization effect in this structure.

A schematic structure of the hybrid MQW LED chip is shown in Fig. 1a. The LED chip is fabricated on a c-plane patterned sapphire substrate (PSS) to enhance the light extraction from the semiconductor materials. When the voltage is applied at the electrodes of the LED chip, a bright yellow-green emission located in the “green gap” range is clearly observed, which brightens rapidly as the injection current increases from 1 to 20 mA, as shown in Fig. 1b. The hybrid MQW LED is then encapsulated and characterized with a calibrated integrating sphere. Excellent LED performance is achieved, as illustrated in Fig. 1c–e. At an injection current of 20 mA, the dominant wavelength is 560 nm, with a high luminous efficiency of 19.58 lm/W. The forward voltage is 3.39 V, which is a normal value for lighting applications. The blue emission from the four low-indium-concentration QWs is negligible in the electroluminescence (EL) spectrum of the hybrid MQW LED, indicating that the high-indium-concentration QW on the top consumes a majority of the injected carriers because the electron mobility is considerably higher than that of the holes.

Another two green LED chips, one with a hybrid active region and the other with a conventional active region, are fabricated on c-plane sapphire substrates and comparatively studied to investigate the light output enhancement of the new structure. Their peak wavelengths are adjusted to approximately 540 nm because measurement of the conventional LED becomes difficult above 550 nm due to its diminished light efficiency. The hybrid MQW LED has the same epitaxial structure as the 560-nm LED, except for a lower indium molar concentration in the top QW. For the conventional green LED, the active region is a four-period MQW structure consisting of 3 nm green InGaN QWs separated by 14 nm GaN barriers. The remaining characteristics of the two LEDs are identical. The structures are confirmed by high-resolution X-ray diffraction (HRXRD) measurements using ω-2θ scans along the growth direction, as shown in Fig. 2a. With the four blue QWs in addition to the green QW, the average indium molar concentration is lower in the hybrid MQWs, which benefits the crystal quality in the top InGaN QW layer. Fig. 2b shows the EL spectra at 20 mA, which indicate that the two LEDs have the same emission peak wavelength at 542 nm. The normalized light output power and forward voltage versus injection current are shown in Fig. 2c. A remarkable enhancement of the light output power is achieved by using the hybrid MQWs.

To study the mechanism of the radiative recombination process in the hybrid MQWs, temperature-dependent photoluminescence (TDPL) measurements are carried out for the two samples. Fig. 3a shows the peak energies of the green emission peaks at different temperatures. A clear S-shaped shift behavior of the peak energy is observed for both samples, suggesting a strong localization effect in the InGaN QWs^22^. The band-tail model is used to fit the S-shaped shift curves. The fitted 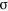, which indicates the degree of the localization effect, is larger for the hybrid MQWs than for the conventional LED, showing a higher density of localized states in the new structure compared with the conventional one. Additional evidence of the stronger localization effects is the difference in the temperature-dependence of the full-width at half-maximum (FWHM) of the two LED samples, as demonstrated in Fig. 3b. As is the case for the peak energy, the FWHMs of both samples exhibit an S-shaped shift with increasing temperature due to the localization effects^23^. Compared to the conventional LED, the FWHM changes more dramatically for the hybrid MQW LED, thus showing a stronger localization effect.

The carrier localization in the InGaN/GaN MQWs is caused by InN-rich InGaN quantum dots (QDs)^24^,^25^ as a result of indium phase separation^26^ and lattice mismatch in the InGaN epilayers with a high indium molar concentration. The existence of the QDs is responsible for the high radiative efficiency despite the high dislocation density in InGaN. In the conventional green LED sample, each QW contains a high-indium-concentration InGaN layer, so a large accumulated stress is expected with the increased stacking of QW layers, and this increased stress causes degradation of the crystal quality in the top QW layer, as also occurs in InAs multilayer QDs^27^. The QDs are inevitably impaired, resulting in the quenching and broadening of the emission peak. Such a problem can be avoided in the hybrid MQWs, where the InGaN QDs in the top QW layer are retained with proper stress because the average indium concentration in the active region is lower. Carriers are preferentially captured by these QDs rather than the defect centers^24^; the QCSE is partially alleviated because the small size of the InGaN QDs prevents the separation of the electron-hole wavefunctions^28^.

The luminous efficiency of 19.58 lm/W achieved in this work is a very high value for c-plane InGaN-based LEDs with normal voltage, compared with previous work on yellow-green LEDs^29^. Our experimental data suggest that the hybrid MQW structure can effectively push the efficient InGaN LED emission toward longer wavelengths, connecting to the lower limit of the AlGaInP LEDs’ spectral range, thus enabling completion of the LED product line covering the entire visible spectrum with sufficient luminous efficacy, which is critical to the expansion of the solid state lighting market. The hybrid MQW structure we demonstrate here yielded higher-efficiency LEDs in the “green gap” range and may also enable InGaN LEDs emitting yellow and even red light, allowing for more flexible and rich choices for the LED colors. In addition, the InGaN-based single-chip white LEDs have been studied for future lighting applications^30^. The color rendering and light efficiency are restricted by the imbalanced light intensity between the blue and longer wavelength emissions. The realization of high-efficiency InGaN active regions emitting in the “green gap” and at longer wavelengths will be a significant step forward for single-chip white LEDs. Additionally, the technique presented here could be applied to produce high-efficiency active regions for other optoelectronic devices, such as solar cells, laser diodes and photon detectors.

In conclusion, we have successfully realized high-luminous-efficiency InGaN LEDs in the “green gap” range. Yellow-green light emission with a dominant wavelength at 560 nm and a luminous efficiency of 19.58 lm/W has been obtained at an injection current of 20 mA. Comparative studies of 540-nm hybrid MQW LEDs and conventional MQW LEDs show that participation of localized states in the radiative recombination in the hybrid MQWs is responsible for the enhancement of the light output power. This work paves the way for InGaN materials with emissions moving toward longer wavelengths, leading to more colorful lighting and more efficient illumination by semiconductor LEDs, and thus demonstrates significant progress in filling the “green gap”.

## Methods

### Material epitaxy

The InGaN/GaN MQW LED samples in this work were grown by the metal-organic chemical-vapor deposition (MOCVD) technique. The MOCVD equipment is an Aixtron 2400G3 system with a planetary reactor chamber. The precursors are trimethylgallium (TMGa), triethylgallium (TEGa), trimethylindium (TMIn), and ammonia (NH3). After annealing the substrate in H_2_ at 1,050 °C, a 30-nm-thick GaN nucleation layer was deposited on the c-plane sapphire substrates at 550 °C. Then, the temperature was ramped to 1,050 °C for annealing and growth of a 3-μm-thick Si-doped GaN layer. The active regions were grown with lower temperatures in a full N_2_ atmosphere, followed by deposition of a 10-nm-thick GaN spacer layer and a 180-nm-thick Mg-doped GaN layer. The conventional LED active region consists of four identical InGaN/GaN green QW pairs, whereas the hybrid MQW LED active region consists of four blue QWs and one green QW. The indium molar concentrations are tuned by adjusting the reactor temperature during the InGaN layer growth. The two comparative samples were grown on planer sapphire substrates, whereas the 560-nm-dominant-wavelength LED was grown on PSS.

### Epi-wafer characterizations

The two comparative epi-wafer samples were characterized by HRXRD and TDPL spectroscopy. HRXRD was performed using a Bede D1 double-axis diffractometer with a parabolic graded multilayer Gutman mirror collimator followed by a four bounce channel-cut Si (2 2 0) monochromator delivering a Cu K_a1_ line with a wavelength of 0.154056 nm. TDPL spectra from 20 to 300 K were recorded using a 405-nm laser diode at an emission power of 60 mW. The emitted light was dispersed by a triple grating monochromator and detected by a GaAs photomultiplier tube using a conventional lock-in technique.

### Band-tail model simulation

The band-tail model^24^ was adapted to fit the TDPL peak energy vs. temperature curves. Normally, the density of localized state (DOS) is assumed to have a Gaussian-shaped distribution such that the temperature (*T*) dependence of the PL peak energy (*E*(*T*)) curve can be fitted by the band-tail model.


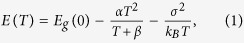


The first two terms of the polynomial are the Varshni empirical equation describing the temperature-induced fundamental bandgap shrinkage of InGaN materials, where *E*_*g*_(0) is the transition energy at 0 K and 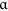 and β are the Varshni thermal coefficients. The third term describes the influence of the localized states, where *k*_*B*_ is the Boltzmann constant and 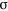 is the standard deviation of the Gaussian distribution and is considered as an indicator of the degree of the localization effect.

### Device fabrication and characterization

After growth, the LED epi-wafers were annealed in N_2_ at 700 °C to activate the Mg-doping to form p-type GaN. The n-type GaN was exposed by inductively coupled plasma (ICP) etching. Ti/Al and ITO were used as n-electrodes and p-electrodes, respectively. The lateral size of an LED chip cell is 350 μm × 350 μm. The two comparative samples were then measured as LED chips using a standard industrial LED testing machine to obtain the normalized light output power, I-V and EL spectra. The 560-nm-dominant-wavelength LED was further cut into individual cells and placed in silicon resin. The output power was measured in a calibrated integrating sphere.

## Additional Information

**How to cite this article**: Jiang, Y. *et al*. Realization of high-luminous-efficiency InGaN light-emitting diodes in the "green gap" range. *Sci. Rep*. **5**, 10883; doi: 10.1038/srep10883 (2015).

## Figures and Tables

**Figure 1:**
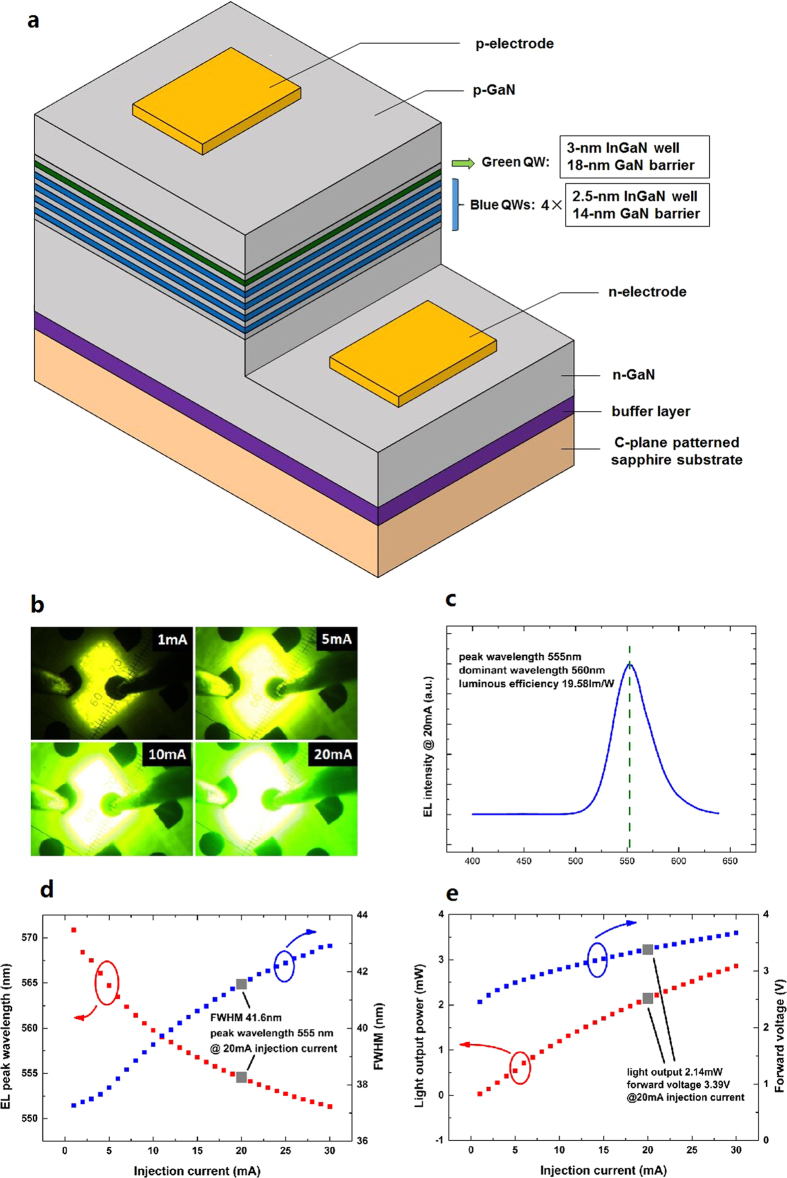
Structure and performance of a yellow-green light-emitting diode (LED) with a dominant wavelength of 560 nm. **a**, Schematic structure of the hybrid multi-quantum well (MQW) LED. The active region is sandwiched between the p-GaN and n-GaN; each of the four blue QWs comprises a 2.5-nm InGaN well layer and a 14-nm GaN barrier layer, whereas the green QW has a 3-nm InGaN layer with a higher indium concentration on top of an 18-nm GaN barrier. **b**, Photographs of the LED chip at different injection currents of 1, 5, 10 and 20 mA at room temperature. **c**, Electroluminescence (EL) spectrum of the encapsulated LED device at 20 mA. A single peak of yellow-green light with a peak wavelength of 555 nm and a dominant wavelength of 560 nm is observed. **d**, EL peak wavelength (red) and full-width at half-maximum (FWHM) (blue) as a function of the injection current. A blueshift (16.4 nm from 1 to 20 mA) of the EL peak occurs due to the quantum-confined Stark effect (QCSE) and band filling. The FWHM of the emission peak is 41.6 nm at 20 mA. **e**, Light output power (red) and forward voltage (blue) as a function of the injection current. At an injection current of 20 mA, the light output power and forward voltage are 2.14 mW and 3.39 V, respectively. The corresponding luminous efficiency is 19.58 lm/W.

**Figure 2:**
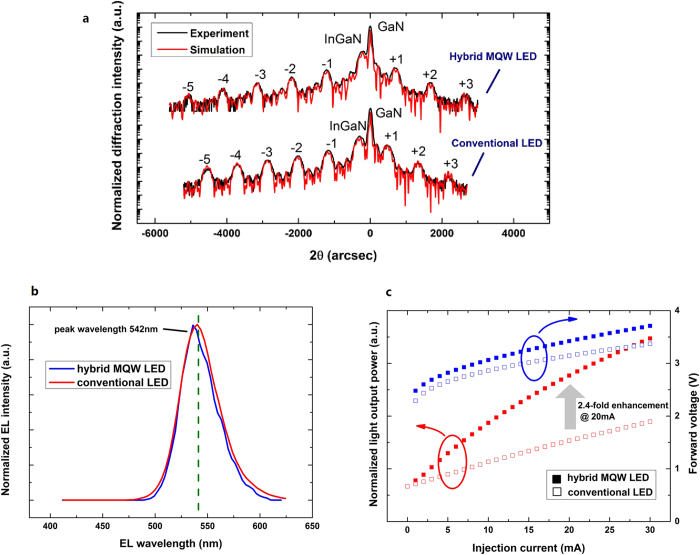
Structures and electroluminescence (EL) properties of green light-emitting diodes (LEDs) with hybrid multi-quantum wells (MQWs) and conventional MQWs. **a**, High-resolution X-ray diffraction (HRXRD) ω-2θ scan curves (black) and simulated curves (red) of the hybrid MQW LED and conventional LED. High-order diffraction peaks of InGaN/GaN structures, where the numbers present different peak orders, can be clearly observed for both samples, indicating that the high-quality periodic MQWs are well formed. The simulation fitting curves, which coincide well with the experimental data, indicate that the indium concentrations in the blue and green QWs are 17% and 30%, respectively. **b**, EL spectra of the hybrid MQW LED (blue) and conventional LED (red) at 20 mA. Single peaks of green light emission centered at the same peak wavelength of 542 nm are obtained from both samples. c, normalized light output power and forward voltage as a function of the injection current for the hybrid MQW LED (solid squares) and the conventional LED (hollow squares). At an injection current of 20 mA, the light output power (red) of the hybrid MQW LED is 2.4-fold higher than that of the conventional LED. The forward voltages (blue) of the hybrid MQW LED and conventional LED are 3.42 and 3.15 V, respectively.

**Figure 3:**
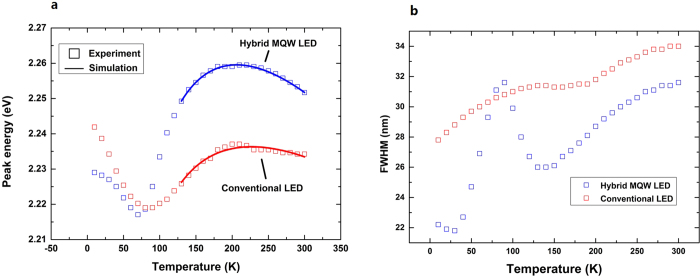
Temperature-dependent photoluminescence (TDPL) results of the hybrid multi-quantum well (MQW) light-emitting diode (LED) and the conventional LED. **a**, PL peak energies of the green emission peaks as a function of temperature for the hybrid MQW LED (blue) and the conventional LED (red). The fitted curves obtained from the band-tail model are plotted with solid lines, and the fitted σ for the hybrid MQW LED and conventional LED are 26.26 and 22.72 meV, respectively. **b**, PL full-widths at half-maximum (FWHMs) of the green emission peaks as a function of temperature for the hybrid MQW LED (blue) and the conventional LED (red). The S-shaped behavior is caused by localized states. When the temperature ramps from 30 K to 90 K, the FWHM increases due to the relaxation of carriers into the localized states. The FWHM then drops when the temperature further increases to 140 K because part of the carriers are captured by nonradiative recombination centers before reaching the lower energy tail states. Above 140 K, the FWHM increases due to coupling of the excitons to acoustic phonons as well as to LO phonons.
